# Early-life infectious and nutritional exposures and cardiovascular risk in early adulthood in Uganda: protocol for a new round of data collection in the Entebbe Mother and Baby Study birth cohort at 21 years (EMaBS@21)

**DOI:** 10.1136/bmjopen-2026-119342

**Published:** 2026-04-07

**Authors:** Isaac Sekitoleko, Ronald Komata, Ivan Ssali, Ronald Kyasanku, Racheal Nakyesige, Moses Sewankambo, Florence Akello, Nelson Twinamasiko, Milly Namutebi, Josephine Tumusiime, Chrisostome Akantorana, Hellen Akurut, Priscilla A Balungi, Mirriam Akello, Sarah H Atkinson, Anxious Niwaha, Anne Wajja, Nambusi Kyegombe, Moffat Nyirenda, Alison M Elliott, Bridgious Walusimbi, Emily L Webb

**Affiliations:** 1 Medical Research Council and London School of Hygiene and Tropical Medicine Uganda Research Unit, Uganda Virus Research Institute, Entebbe, Central Region, Uganda; 2 Department of Infectious Disease Epidemiology, London School of Hygiene & Tropical Medicine, London, UK; 3 Kenya Medical Research Institute (KEMRI) Centre for Geographic Medicine Research Coast, KEMRI-Wellcome Trust Research Programme, Kilifi, Kenya; 4 Centre for Tropical Medicine and Global Health, University of Oxford, Oxford, UK; 5 Department of Paediatrics, University of Oxford, Oxford, UK; 6 Department of Non-Communicable Disease Epidemiology, London School of Hygiene & Tropical Medicine, London, UK; 7 Clinical Research Department, London School of Hygiene & Tropical Medicine, London, UK; 8 Department of Global Health and Development, London School of Hygiene & Tropical Medicine, London, UK

**Keywords:** Cardiovascular Disease, Blood Pressure, Africa South of the Sahara, Longitudinal studies, Nutrition, INFECTIOUS DISEASES

## Abstract

**Abstract:**

**Introduction:**

Non-communicable diseases, particularly cardiovascular diseases (CVDs), have become major contributors to morbidity and mortality in sub-Saharan Africa (SSA) and are projected to surpass infectious diseases as the leading cause of death among adults by 2030. Although CVDs have traditionally been associated with older age and obesity, adverse cardiovascular phenotypes are increasingly being observed in younger and leaner individuals in SSA. This pattern suggests that pathways to CVD risk in SSA may differ from those described in high-income countries. Early-life infectious exposures, undernutrition and socio-demographic conditions common in many SSA settings have been proposed as potential risk factors. Still, empirical evidence linking these exposures to cardiovascular risk in early adulthood remains limited due to a scarcity of long-running birth cohorts in the region.

**Methods and analysis:**

This protocol describes a new round of data collection nested within the Entebbe Mother and Baby Study (EMaBS), a population-based Ugandan birth cohort established originally as a clinical trial (ISRCTN32849447) between 2003 and 2006 with prospective follow-up from pregnancy through adolescence. All participants currently under follow-up will be invited to participate at approximately 21 years of age. Primary outcomes are physiological determinants of CVD measured in early adulthood, including blood pressure, blood lipid levels, body mass index, body composition and markers of glucose metabolism. Secondary outcomes include behavioural CVD risk factors (diet, physical inactivity, alcohol use and tobacco use) and qualitative measures of CVD knowledge and risk perception. Key exposures of interest include prospectively collected early-life and childhood infectious exposures (malaria and helminth infections), markers of growth and undernutrition, micronutrient status, inflammatory markers, socio-demographic factors and selected genetic variants. Quantitative analyses will use multivariable regression and causal modelling approaches and will be complemented by qualitative interviews and focus group discussions.

**Ethics and dissemination:**

The study protocol has been reviewed and approved by the Uganda Virus Research Institute Research and Ethics Committee (UVRI REC Ref: GC/127/35), the Uganda National Council for Science and Technology (UNCST Ref: MV625), and the London School of Hygiene & Tropical Medicine Research Ethics Committee (LSHTM Ethics Ref: 8811). Written informed consent will be obtained from all participants before study activities. Study findings will be shared and discussed with participants and community stakeholders through established engagement platforms. Results will be disseminated to the scientific community through peer-reviewed publications and conference presentations, and data will be made available to other researchers via established data-sharing platforms. We will engage policymakers at the district, national and international levels to facilitate the translation of findings into policy-relevant outputs.

**STRENGTHS AND LIMITATIONS OF THIS STUDY**

- This study builds on one of the longest-running and largest birth cohorts in low-income sub-Saharan Africa, with prospective data collected from the prenatal period through early adulthood.
- The cohort includes detailed longitudinal data on nutritional, infectious, growth and socio-demographic exposures.
- In addition to stored biological samples and genetic and epigenetic data, the longitudinal data of the cohort will be expanded with this new round of data collection.
- Due to strong participant and community engagement, the cohort has maintained support for long-term participant retention.
- One potential challenge is achieving the target sample size, given that recent nested studies within the Entebbe Mother and Baby Study have involved smaller participant groups, which may potentially lead to selection bias.

## Introduction

 Non-communicable diseases (NCDs), including cardiovascular diseases (CVDs), diabetes and cancer, are the leading cause of death globally. In sub-Saharan Africa (SSA), they are projected to overtake infectious diseases as the primary cause of adult mortality by 2030,^1^ with CVDs emerging as the leading contributor. This rising burden of CVDs has been largely attributed to the epidemiological transition, increased longevity and increased exposure to modifiable behavioural risk factors such as unhealthy diet (ie, high in salt, fat and free sugars, and low in fruit and vegetables), physical inactivity, harmful alcohol use and tobacco use.^2^ Although these conditions have been previously observed predominantly among the elderly and non-lean individuals, CVDs are increasingly occurring at younger ages and at lower levels of adiposity in SSA.^3^ This suggests that pathways to CVD risk in SSA may differ from those described in high-income countries, in which these conditions generally manifest later in life and are closely linked to adult lifestyle factors.

The causes of these observed differences remain unknown, but a role for genetics, and for early-life infectious exposures, and undernutrition, which are common in many African countries and often distinct from high-income country populations, has been proposed.^4^ These distinct early exposures may drive different physiological pathways to CVD risk.^5^ Previous studies have demonstrated that important determinants of CVDs begin early in life, with cardiovascular and metabolic changes seen in childhood and adolescence, long before clinical manifestation of CVD in adulthood.^6^ Progress in understanding these pathways in SSA has been constrained by the limited availability of longitudinal studies with detailed life-course data on nutrition, growth, infectious and socio-demographic exposures, and long-term follow-up into adolescence and early adulthood. Although several African birth cohorts have been established, to our knowledge, only one cohort in urban South Africa has investigated associations between early-life and life-course exposures (focusing on sociodemographics and growth) and CVD-related outcomes in late adolescence.^7^

The Entebbe Mother and Baby Study (EMaBS) is a population-based Ugandan birth cohort with extensive socio-demographic, genetic, anthropometric, infection, immunisation, nutrition and immune response data from the prenatal period through to adolescence, together with stored samples from multiple time points across the life course.^8 9^ Data are also available on physiological determinants of CVDs measured at the start of adolescence at 10–11 years.^10^

Between 2024 and 2027, EMaBS participants will reach 21 years of age. This is approaching the end of adolescence, a time of rapid growth and physiological change/turbulence triggered by pubertal hormonal changes, and a time during which behaviours emerge that will determine health in later adulthood; approximately 70% of premature deaths during adulthood are estimated to be the result of health-related behaviours initiated by the end of adolescence.^11^ Assessment at this age, therefore, allows evaluation of key behavioural risk factors initiated during adolescence, including knowledge and understanding of these risk factors, and measurement of physiological determinants of CVDs, which will track into later adulthood.^6^ The EMaBS cohort thus offers a unique opportunity to explore the hypotheses regarding the impact of early-life infectious, nutritional and socio-demographic exposures characteristic of SSA on adult CVD susceptibility.

### Study hypothesis and objectives

The primary hypothesis is that early-life infectious exposures and undernutrition, common in many African settings, influence physiological determinants of CVD measured in early adulthood. Secondary hypotheses are that early-life socio-demographic factors influence knowledge and awareness of CVDs and initiation of risk behaviours for CVDs during adolescence, and that these are associated with physiological determinants of CVDs in this setting.

**Figure 1:**
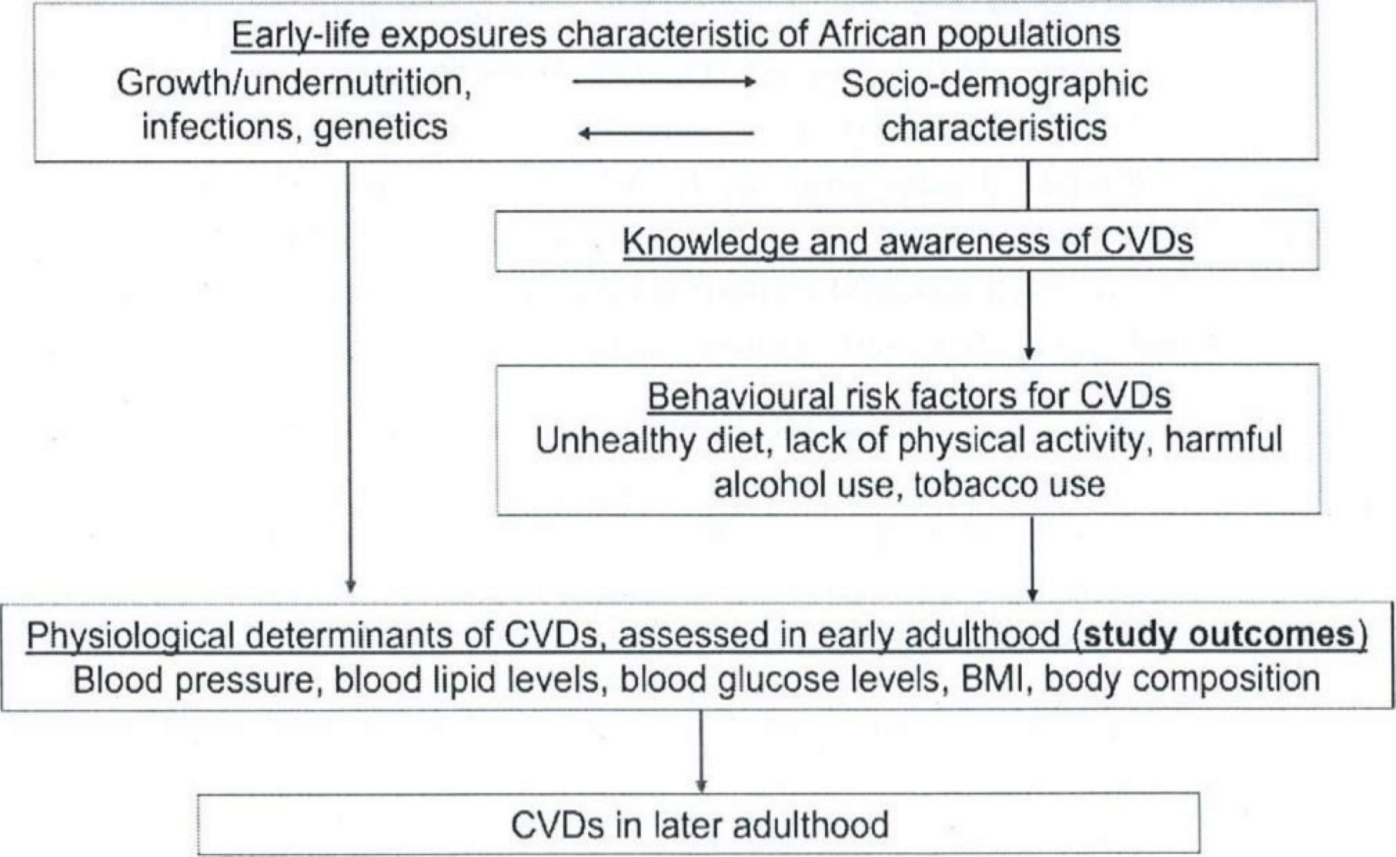
Conceptual framework for the Entebbe Mother and Baby Study at 21 year. BMI, body mass index; CVD, cardiovascular disease.

The study objectives are to: (1) determine the effect of early-life exposures on physiological determinants of CVD in young Ugandan adults; (2) (a) identify determinants of behavioural CVD risk factors and (b) examine their associations with physiological CVD determinants in this population; (3) investigate nutritional and metabolic pathways linking early-life exposures to adult physiological CVD risk; and (4) explore the understanding of CVD risk among participants, including awareness of behavioural risk factors and sources of information on NCDs. In this study, physiological determinants of CVD are defined as blood pressure, blood glucose, insulin, blood lipid levels, body mass index (BMI), and body composition. Behavioural risk factors for CVD include unhealthy diet, physical inactivity, harmful alcohol use and tobacco use (figure 1).

## Methods and analysis

### Planned start and end dates for the study

The planned start date for this study is 22 October 2024, and the planned end date is 31 March 2027.

### Study design

This is a new round of data collection in a longitudinal study. It will leverage and extend the existing EMaBS birth cohort. The participant data collected in this new round will be linked to earlier individual data collected from pregnancy through childhood to date. The new round of data collection began on 22 October 2024, and is currently ongoing.

### Patient and public involvement statement

Involvement of the participants and the broader community has been fundamental to study preparation, enrolment and ongoing follow-up. During the planning stages of the study, meetings were held with community leaders to explain the planned research questions and obtain input into the conduct of the study. Each village in the study area has an elected committee, and at the start of EMaBS, each of these committees appointed volunteer assistants (‘community field workers’), members of the public who have played a key role in the study. They continue to provide valuable support to EMaBS through follow-up and engagement activities. More recently, the Medical Research Council/Uganda Virus Research Institute and London School of Hygiene & Tropical Medicine (MRC/UVRI and LSHTM) Uganda Research Unit has established a community advisory board, which has also been engaged in meetings to discuss this work in the cohort. This has been complemented by meetings with participants and their parents and caregivers to obtain their input into the project plans.

### Study populations and recruitment

The study will be conducted among EMaBS participants, who will be contacted and invited to participate in Entebbe Mother and Baby Study at 21 years (EMaBS@21). The EMaBS birth cohort began life as a randomised controlled trial aimed at investigating the impact of anthelmintic treatment during pregnancy and early childhood on responses to immunisations, incidence of infectious and allergy-related diseases, and growth and nutrition (ISRCTN32849447).^9^ A total of 2345 documented live-born children were recorded in the cohort between 2003 and 2006. EMaBS cohort attrition due to mortality or loss to follow-up was highest during the first 5 years of life (27% in infancy, 45% from birth to 5 years) and has stabilised at an average of ~2% per year since then. For example, 1270 participants were seen at age 5 years, 1119 at age 10–11 years and 976 were seen at the EMaBS clinic when aged 15–18 years. In April 2023, we undertook a cohort census in concert with a dissemination open day. A total of about 1500 cohort participants, over half of whom attended the open day, have provided comprehensive location and contact details and expressed willingness to be contacted to take part in future waves of data collection. We will use a census-based sampling approach for this study, that is, we plan to sample as many EMaBS participants as are available and meet the eligibility criteria within the recruitment time frame.

### Inclusion and exclusion criteria

Inclusion criteria for the proposed data collection are: (1) being an EMaBS participant, (2) aged 18 or above, and (3) able to provide written and informed consent before taking part in this study. Potential participants who are clinically unwell will be asked to return when they have recovered. Exclusion criteria are current pregnancy, having been pregnant within the last 6 months, or already having a diagnosis of CVD or diabetes and being on medication (and hence physiological measures are influenced by this medication).

### Outcomes and exposures

The primary outcomes are physiological determinants of CVD measured in early adulthood, including systolic and diastolic blood pressure, blood lipid concentrations (total cholesterol, high-density lipoprotein cholesterol, low-density lipoprotein cholesterol and triglycerides), BMI, body composition measures (including fat mass and fat-free mass) and markers of glucose metabolism (fasting blood glucose, glycated haemoglobin (HbA1c) and insulin levels). Secondary outcomes include behavioural CVD risk factors, including unhealthy diet, physical inactivity, harmful alcohol use, current tobacco use and poor sleep, assessed using structured questionnaires. Qualitative outcomes include the understanding of CVDs, awareness of behavioural risk factors, perceived personal risk and sources of information on NCDs among participants.

The primary exposures of interest differ by objectives, as shown below.

1. For objectives 1 and 2a, exposures include early-life infectious exposures (malaria and helminth infections, including *Schistosoma mansoni* and soil-transmitted helminths, measured repeatedly from in utero through childhood), markers of early-life growth and undernutrition (birth weight and growth trajectories), micronutrient status in early childhood (iron indices, vitamin D, vitamin A, vitamin E, zinc, vitamin B12 and folate), inflammatory markers (C-reactive protein) and early-life socio-demographic characteristics (parental education, household socio-economic status and home environment indicators). Selected genetic variants derived from whole-genome data generated from samples collected in early childhood will be considered as secondary exposures.
2. For objective 2b, behavioural risk factors assessed in adolescence and early adulthood (diet, physical activity, alcohol use, tobacco use and sleep quality) will be treated as exposures in analyses examining associations with physiological CVD determinants.
3. For objective 3, analyses will focus on nutritional, infectious, metabolomic and socio-demographic pathways linking early-life exposures to physiological CVD risk in early adulthood. Findings from objective 1 will inform candidate pathways that will include longitudinal infection data, early-life micronutrient and inflammatory markers, metabolomic profiles derived from archived and newly collected samples, and early-life socio-economic conditions.

### Data collection

Participants will be invited to attend the study clinic once during the project period, at or near age 21 years. We will facilitate visits to the study clinic for participants residing both within and outside the original study area. Participants will attend two study visits. At the first visit (day 1), screening procedures will be conducted and written informed consent will be obtained. This visit will allow adequate time to explain the study procedures, including the fasting requirement, and to address any participant questions. The second visit (day 2) will involve the main study procedures and will be conducted after an overnight fast. Fasting blood samples will be obtained at the beginning of this visit, after which participants will be provided with a meal and refreshments as soon as possible.

We will collect data through questionnaires and physical examination. Questionnaires will be used to update existing socio-demographic information for each participant, collect information on current living conditions, family composition, level of education attained and occupation, and collect information on behavioural risk factors for NCDs, including physical activity, diet, alcohol use, tobacco use, mental health, sleep quality and well-being. Tobacco use will be assessed through self-report, including current and previous smoking status, and number of pack-years. Alcohol use will be assessed through the 30-day Alcohol Use Disorders Identification Test (AUDIT-40), which has been validated against the Timeline Follow Back method, and against phosphatidylethanol in blood for detecting harmful alcohol use in Ugandan youth.^12^ Physical activity will be assessed using the WHO Global Physical Activity Questionnaire, which has been shown to have moderate to high validity and reliability across 12 countries worldwide and was previously used in a Ugandan countrywide survey.^13^ Diet and nutrition will be assessed through food frequency questionnaires, which will include commonly consumed food items from the Uganda regional food surveys; energy and nutrient content will be calculated from available food composition tables; we will focus on high intake of sugar, salt and fat, low intake of fruits and vegetables, and high intake of processed foods. Common mental health disorders will be assessed using the Patient Health Questionnaire−9^14^ and the generalised anxiety disorder−2.^15^ Sleep quality will be assessed using the insomnia severity index.^16^

The physical examination will include measurements of height, weight, waist circumference, blood pressure and body composition. Participants will be measured wearing light clothing and without shoes. Height will be measured to the nearest centimetre, and weight to the nearest 0.1 kg. Waist circumference will be measured at the mid-way position between the lowest rib and the iliac crest and recorded to the nearest centimetre. Body composition will be measured using a Tanita body composition analyser (MC-580M S, TANITA Corporation, Tokyo, Japan). Trained nurses will measure blood pressure three times, 3 min apart, using an appropriately sized cuff on the right arm, supported at heart level, with the participant seated upright all the way to the back of the chair, legs uncrossed and feet flat on the floor. Automated Omron (Kyoto, Japan) (M6, HEM-700) machines validated every 6 months by the Uganda National Bureau of Standards will be used. The mean of the second and third measurements will be used for analysis. We will also obtain fasting blood (as early in the visit as possible), stool samples and urine (only from female participants for pregnancy testing at screening).

To better understand social and behavioural factors that may influence the risk of CVD in individuals and collect initial data on the nature of acceptable interventions, we will also conduct 50 qualitative in-depth interviews and five focus group discussions. Participants for the in-depth interviews and focus group discussions will be purposively sampled from those who are enrolled in the study. We will use maximum variability sampling to obtain a heterogeneous sample. All interviews will be audio-recorded with consent and guided by a semi-structured topic guide.

### Laboratory methods

Lipid parameters will be measured in fasting blood serum using a homogeneous enzymatic colourimetric assay. Blood glucose and insulin sensitivity will be measured in fasting venous blood samples, with insulin sensitivity estimated using the Homeostasis Model Assessment of Insulin Resistance, and HbA1c will be measured using a turbidimetric inhibition immunoassay. All biochemical analyses will be performed using a Roche (Roche Diagnostics Ltd, Burgess Hill, United Kingdom) Cobas 6000 platform (c 501 module) at the Clinical Diagnostics Laboratory based at MRC/UVRI and LSHTM Uganda Research Unit. A portion of the blood sample will be used for a complete blood count and examined for malaria by PCR and for *S. mansoni* by circulating anodic antigen. The rest of the blood will be used for peripheral blood mononuclear cell separation to allow for investigation of cellular immune responses.

Stool samples will also be collected from the participants to enable measurement of helminth infections using PCR, and for investigation into the link between the gut microbiome and CVD risk in a longitudinal framework. Among subgroups of participants selected based on phenotypic extremes (exposure and outcome), further detailed phenotyping will be undertaken, guided by which early-life exposures are associated with which physiological determinants of CVDs, to investigate potential pathways for these associations. Based on our earlier data showing that biological pathways associated with cholesterol metabolism are significantly enriched in *S. mansoni*-infected participants compared with uninfected participants,^17^ we plan to extract metabolites from new serum samples collected from participants and from stored samples collected in childhood. Metabolites will be profiled using liquid chromatography mass spectrometry at the Biological Mass Spectrometry Core Research Facility at the University of Manchester. Blood and serum samples will be stored to enable future work, including epigenetic studies, to be designed based on the findings of the proposed work.

### Data management

Data will be captured electronically and managed using Research Electronic Data Capture (REDCap) electronic data capture tools hosted at MRC/UVRI. All data, including audio recordings, will be anonymised and stored on a secure and password-protected server, with access limited to essential research personnel. Data and samples will be labelled with the unique identification number of each participant to enable linkage with historical data. To ensure data quality and reliability, clinical and laboratory procedures will follow standard operating procedures. Range checks will be programmed into data collection tools in REDCap. The study will be regularly monitored and conducted in accordance with Good Clinical Practice and Good Clinical Laboratory Practice principles. Qualitative interviews will be audio-recorded using an encrypted digital recording device that is kept on the researcher’s person at all times. Audio files will be uploaded to a secure server as soon as possible after the interview and thereafter deleted from the audio device. No identifying information will be recorded. Audio recordings will be transcribed verbatim and stored on a secure server. When in use, transcripts will be analysed on an encrypted laptop, either in the custody of a researcher or in a safe location at the MRC/UVRI and LSHTM Uganda Research Unit offices.

### Sample size determination

Our target minimum sample size is 900 participants. Our preliminary data suggest that blood pressure will be normally distributed with SD in the range 8–10 mmHg, and that lipid parameters will be normally distributed (or amenable to normalisation by log-transformation) with SD in the range 0.4–0.8 mmol/L. Our key early-life exposures of interest will have variable distributions and prevalence. Table 1 shows mean differences in outcome between exposed and unexposed groups that we will have 80% power to detect at a 5% significance level across a range of exposure prevalence, with adjustment for confounders.

**Table 1:** Power estimates for the effect of exposures on primary outcomes (objectives 1 and 2)

	SD for blood pressure outcomes, mmHg					SD for lipid parameter outcomes, mmol/L				
Exposure prevalence	8	8.5	9	9.5	10	0.4	0.5	0.6	0.7	0.8
5%	3.7	3.9	4.2	4.4	4.6	0.19	0.23	0.28	0.32	0.36
10%	2.8	2.9	3.1	3.3	3.4	0.14	0.18	0.21	0.24	0.27
20%	2.2	2.3	2.4	2.5	2.6	0.12	0.14	0.16	0.18	0.21
30%	1.9	2.0	2.1	2.2	2.3	0.11	0.12	0.14	0.16	0.18
40%	1.8	1.9	2.0	2.1	2.2	0.10	0.12	0.13	0.15	0.17
50%	1.8	1.9	2.0	2.1	2.2	0.09	0.11	0.13	0.15	0.17

No formal adjustment for multiplicity is planned; we shall analyse a pre-specified set of exposures and outcomes and focus on patterns and consistency of results. The target sample size ensures sufficient power to detect small-to-moderate differences in mean outcome (<4 mmHg for blood pressure (BP) and <0.3 mmol/L for lipid parameters).

### Statistical analysis

All quantitative analyses will be conducted using Stata (v18, StataCorp, College Station, Texas, USA) and R (v4.5, R foundation for Statistical Computing, Vienna, Austria). Analyses will be both descriptive and inferential, using multivariable regression models with appropriate adjustments for confounders. Participant recruitment, follow-up and exclusions will be illustrated using a flow diagram, in line with the Strengthening the Reporting of Observational Studies in Epidemiology (STROBE) guidelines for observational studies. Descriptive statistics will be used to summarise baseline characteristics, means and SD for normally distributed continuous variables, medians and IQRs for skewed distributions, and frequencies and percentages for categorical variables. The characteristics of EMaBS participants included in EMaBS@21 will be compared with the characteristics of EMaBS participants not included in EMaBS@21 to determine how generalisable the findings are to the originally recruited cohort of participants. Characteristics to be compared will be those collected during pregnancy at the original EMaBS enrolment at delivery and during childhood (for those followed to this period).

#### Objective 1: to determine the effect of early-life exposures on physiological determinants of CVDs measured in young Ugandan adults

We will use regression analysis to assess associations with our primary exposures of interest—namely infections and growth/undernutrition—across specific life-course exposure periods. For continuous outcomes, we will use linear regression models to assess associations between each exposure and each outcome, adjusting for confounders. For binary outcomes, logistic regression models will be used to determine the effect of each exposure on the outcome, adjusting for confounders. First, exposures will be modelled individually at each time point to examine cross-sectional associations with the outcomes. Where exposure data have been collected repeatedly over time, such as for malaria and *S. mansoni* infections, we will explore trajectories of exposure and their relationships with study outcomes using latent class or trajectory analysis. Repeated binary measures will also be analysed as time-varying covariates in mixed-effects models.

Additionally, we will leverage longitudinal data collected on the same outcome measures at age 10–11 years, which will also enable the inclusion of participants with some missing time-point data. Causal modelling approaches will be used to obtain unbiased estimates of the average causal effect of each early-life exposure on each outcome, adjusting for confounders. For each exposure-outcome relationship, we will construct directed acyclic graphs to identify the minimal sufficient set of confounders. Inverse probability weighting will be applied to create a pseudo-population in which exposure is independent of measured confounders, thereby enabling adjustment for selection bias due to differential loss to follow-up or missing outcome data. We will also explore secondary exposures of interest, including selected genetic variants from existing whole-genome data that have been linked to CVD risk in other populations.

#### Objective 2: to assess (a) determinants of behavioural risk factors for CVDs, and (b) their associations with physiological determinants of CVDs in young Ugandan adults

For objective 2a, early-life and life-course predictors of behavioural risk factors such as tobacco use, alcohol use, physical inactivity and unhealthy diet will be explored. Logistic regression models will be used for tobacco and harmful alcohol use, and ordered logistic regression will be used to model levels of physical activity. To characterise the diet, principal components analysis will be applied to derive dietary patterns, and Least Absolute Shrinkage and Selection Operator models may also be used to improve robustness. For objective 2b, the behavioural risk factors will serve as exposures and physiological determinants as outcomes, analysed using linear regression.

### Objective 3: investigate nutritional and metabolic pathways through which early-life exposures may influence physiological determinants of CVDs

We will focus on both nutritional and metabolomics pathways. We will examine whether early-life growth influences micronutrient levels before age 5 years and whether these, alongside inflammation biomarkers, are associated with physiological risk markers at age 21 years. For metabolomics, we will analyse samples from individuals at the extremes of phenotypic expression. Metabolites will be identified and quantified using Progenesis QI software (Waters Corporation, Milford, MA, USA), and pathway-level analyses will be conducted using Integrated Molecular Pathway Level Analysis (IMPaLA). We will explore how early-life metabolic profiles relate to profiles at age 21 years and to extremes in exposure (eg, repeated *S. mansoni* infection vs none) and outcomes (eg, high vs low blood pressure). Causal mediation analysis will estimate both direct and indirect effects using a counterfactual framework, accounting for multiple mediators and applying sensitivity analyses to evaluate robustness to potential violations of confounding assumptions and measurement error.

Missing data will be assessed and handled using multiple imputation by chained equations when over 5% of data are missing for key variables, where applicable. Where repeated-measures data are available and intermediate data points are missing, we will use multiple imputation by chained equations to impute missing values, leveraging information from previous and subsequent measurements as well as relevant covariates. Therefore, imputations will include outcome variables and predictors of missingness, with 50 imputed datasets combined using Rubin’s rules.

### Ethics and dissemination

The study protocol has been reviewed and approved by the UVRI Research and Ethics Committee (UVRI REC Ref: GC/127/35), the Uganda National Council for Science and Technology (UNCST Ref: MV625), and the LSHTM Research Ethics Committee (LSHTM Ethics Ref: 8811). Any protocol amendments will be submitted for review and approval. Informed written consent will be obtained from all participants before study activities and will include provision and discussion of detailed information on what participation would involve, so that participants can make an informed decision based on their own perception of the risks and benefits. Participants will be compensated for their time and any incurred travel costs. For participants identified as having conditions that require long-term management, such as high BP or abnormal glucose or lipid levels, study clinicians will provide initial management and information on non-pharmacological management and refer to the hospital for further care. Participants identified as having conditions amenable to immediate management will be treated by the study clinicians. Focus group discussions carry the potential risk of confidentiality breaches if information is shared outside the groups. To mitigate this, we will continuously emphasise the importance of confidentiality to study participants throughout the study period.

Dissemination and stakeholder engagement are integral to the study design. Plans and findings will be shared and discussed with participants and community stakeholders through established engagement platforms, including participant advisory groups and locally appropriate communication channels. Results will be disseminated to the scientific community through peer-reviewed publications and conference presentations, and data will be made available to other researchers via established data-sharing platforms. Engagement with policymakers at district, national and international levels will facilitate translation of findings into policy-relevant outputs, with particular emphasis on informing NCD strategies in Uganda and the wider region.

## Data Availability

Data sharing not applicable as no datasets generated and/or analysed for this study.
